# Association between frailty and ischemic heart disease: a systematic review and meta-analysis

**DOI:** 10.1186/s12877-021-02304-9

**Published:** 2021-06-10

**Authors:** Rosa Liperoti, Davide L. Vetrano, Katie Palmer, Tomasz Targowski, Maria C. Cipriani, Maria R. Lo Monaco, Silvia Giovannini, Nicola Acampora, Emanuele Rocco Villani, Roberto Bernabei, Graziano Onder, on behalf of ADVANTAGE JA WP4 group

**Affiliations:** 1 Università Cattolica del Sacro Cuore, Fondazione Policlinico Universitario A. Gemelli IRCCS, Largo F. Vito 1, 00168 Rome, Italy; 2 Fondazione Policlinico Universitario A. Gemelli IRCCS, Rome, Italy; 3 Aging Research Center, Department of Neurobiology, Health Care Sciences and Society, Karolinska Institutet and Stockholm University, Stockholm, Sweden; 4 Department of Geriatrics, National Institute of Geriatrics, Rheumatology, and Rehabilitation, Warsaw, Poland; 5 Istituto Superiore di Sanità, Rome, Italy

**Keywords:** Frailty, Ischemic heart disease, Systematic review, Metanalysis

## Abstract

**Background:**

Frailty is increasingly reported among older adults with cardiovascular diseases and it has been demonstrated to increase negative health outcomes and mortality. To date, no systematic review of the evidence is available regarding the association between frailty and ischemic heart disease (IHD). We performed a systematic review of literature and a meta-analysis to assess the association between frailty and IHD.

**Methods:**

We selected all the studies that provided information on the association between frailty and IHD, regardless of the study setting, study design, or definition of IHD and frailty. PubMed, Web of Science and Embase were searched for relevant papers. Studies that adopted the Fried definition for frailty were included in the meta-analyses. For each measure of interest (proportions and estimates of associations), a meta-analysis was performed if at least three studies used the same definition of frailty. Pooled estimates were obtained through random effect models and Mantel-Haenszel weighting.

**Results:**

Thirty-seven studies were included. Of these, 22 adopted the Fried criteria to define frailty and provided estimates of prevalence and therefore they were included in meta-analyses. The pooled prevalence of IHD in frail individuals was 17% (95% Confidence Interval [95%CI] 11–23%) and the pooled prevalence of frailty in individuals with IHD was 19% (95% CI 15–24%). The prevalence of frailty among IHD patients ranged from 4 to 61%. Insufficient data were found to assess longitudinal association between frailty and IHD.

**Conclusions:**

Frailty is quite common in older persons with IHD. The identification of frailty among older adults with IHD should be considered relevant to provide individualized strategies of cardiovascular prevention and care. Further research should specifically explore the association between frailty and IHD and investigate the potential common biological ground.

**Supplementary Information:**

The online version contains supplementary material available at 10.1186/s12877-021-02304-9.

## Background

Frailty is a clinical syndrome that occurs in some older adults, which makes the individual vulnerable to stressors. The condition is the result of an impaired homeostatic reserve of the organism coupled with biological damages that occur during aging [1, 2]. Several operational definitions are available to measure frailty [3]. One of the most frequently used definitions in clinical research is the “frailty phenotype” provided by Fried and colleagues [4] According to the Fried criteria, the frailty phenotype is defined as the presence of at least three of the following conditions: unintentional weight loss, self-reported exhaustion, slow gait speed, low energy expenditure and weak grip strength. The frailty phenotype has often been modified by researchers, and these modifications have an important impact on its classification and predictive ability [5].

Frailty is frequent in the geriatric population. Independently of the operational definition adopted to identify frailty, it has been estimated that over 10% of community dwelling older adults and nearly half of the nursing home residents are frail [6, 7]. Compared with non-frail, or “robust”, individuals of the same age, those who are frail show a higher risk of negative clinical outcomes, hospitalization, institutionalization, disability and death [8].

Frailty is common in older adults with cardiovascular diseases [9]. In these patients, frailty status appears to influence the prognosis of the cardiovascular disease and increase mortality [4]. The identification of frailty is increasingly considered relevant for physicians to stratify the cardiovascular risk, to make diagnostic and therapeutic choices and to personalize the pharmacological and non-pharmacological disease management [10–12]. Even if there are no guidelines available on which frailty definition is more suitable when dealing with cardiovascular disease, the Fried phenotype can estimate a larger effect size than a combined Fried/non-Fried frailty assessment for the end point of mortality [13].

A common biological ground for frailty and several cardiovascular diseases has been hypothesized [10]. For example, the release of inflammatory circulating cytokines in response to cellular processes such as increased oxidative stress, DNA damage and mitochondrial dysfunction have been demonstrated to occur in both frailty and heart failure and may represent the pathophysiological link between these two conditions [11]. Similarly, chronic systemic inflammation and insulin resistance, which are involved in the in atherosclerosis and its complications, may contribute to the accumulation of damages in the musculoskeletal and metabolic systems, leading to the development of frailty [12].

There is increasing evidence – though sometimes contrasting – of a possible association between frailty and cardiovascular diseases such as hypertension, atrial fibrillation and heart failure [14, 15]. To date, the available evidence on the association between frailty and ischemic heart disease (IHD) has only partly been summarized, regarding acute coronary syndrome (ACS), that is an acute manifestation of IHD [16] Since frailty is independently related to strong outcomes such as all-cause mortality [17] and readmissions [18] in elderly patients with ACS, its assessment should be integrated into the current existing management to help physicians to applicate appropriate management strategies. Still, there is no available evidence on the magnitude of the association between frailty and IHD.

## Methods

The aim of the present study was to conduct a systematic review of the literature to provide pooled estimations of the evidence regarding the association between frailty and IHD.

We reviewed studies providing information on the association between frailty and IHD in adult subjects (i.e. age > 60 or average age > 60), regardless of the study setting, study design, or definition of IHD and frailty. The protocol of the present study was registered in the international prospective register of systematic reviews PROSPERO (registration number 58303). This systematic review was carried out in accordance with the Preferred Reporting Items for Systematic Reviews and Meta-Analyses (PRISMA) recommendations [19].

### Data sources and searching

We searched three databases for relevant articles published from Jan 1st, 2002 to January 31st, 2020: 1) PubMed electronic database of the National Library of Medicine, 2) Web of Science and; 3) Embase. MeSH terms and free words referring to frailty and IHD were used as keywords. The detailed search queries are reported in the Appendix. References from the selected papers and from other relevant articles were screened for potential additional studies.

### Study selection and data extraction

Two assessors screened independently the title and abstract of the selected studies. The inclusion criteria were: 1. papers reporting information on the association of frailty with IHD; 2. papers in English or another European language; 3. study design: cross-sectional, case-control, or cohort studies. Papers were excluded if they 1. did not report the association between frailty and IHD; 2. included persons younger than 18 years; 3. did not report original data (e.g., editorial, review, or congress abstract); 4. did not provide an explicit definition of frailty and; 5. assessed frailty only with a single symptom/measure (e.g. only gait speed or grip strength); 6. were not in English or another European language. The full text of the papers selected by one or more of the assessors were retrieved for full evaluation. Two assessors read the full texts and independently extracted the information from the selected studies. A third assessor reviewed the data extraction, and any disagreement was resolved through consensus.

### Assessment of risk of bias

Quality of the studies was evaluated independently by the two assessors through the tool for the qualitative evaluation of observational studies Newcastle Ottawa Scale (NOS) [20]. Any disagreement in quality assessment was resolved through consensus. Studies scoring > 7 were considered at low risk of bias, scores of 5–7 indicated moderate risk of bias, and scores of < 5 indicated high risk of bias.

### Statistical analysis

For each measure of interest (i.e. proportions and estimates of associations), a meta-analysis was performed if at least three studies used the same definition of frailty. Only studies that adopted the Fried operational definition for frailty were included in the meta-analyses to increase homogeneity of pooled studies [4]. Considering the observational design of the retrieved studies, and the methodological differences potentially responsible for a significant share of the variance within the measures of interest, the pooled estimates were obtained through random effect models and Mantel-Haenszel weighting. Lack of homogeneity within the pooled studies was assessed through the I^2^ statistics (significant if ≥50%). Publication bias was assessed by mean of the Egger’s and the Begg’s tests. All statistical analyses were performed with STATA version 14 (StataCorp, TX, USA). A *P* value < 0.05 was considered statistically significant.

## Results

**Fig. 1:**
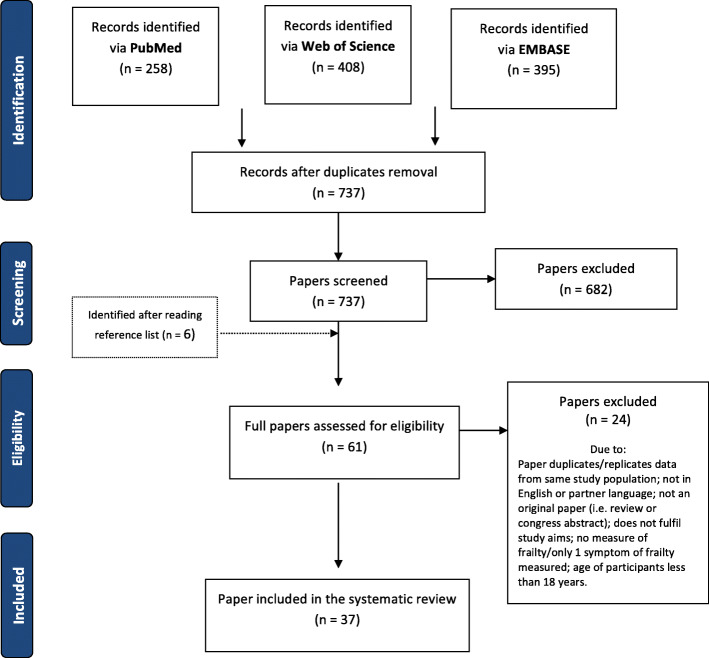
PRISMA flow-chart

The PRISMA flow chart, including the key words used in the search strategies, the number of papers identified, and the number and reasons for excluding papers at each stage is reported in Fig. 1.

Through the literature search, we retrieved 1061 papers. Out of these, 737 were screened after duplicates were removed. Additional 6 studies were identified after reading references from the selected papers. Of the resulting 743 papers, 682 were excluded after screening and 24 after full-text reading. Thirty-seven papers were part of the final qualitative assessment. Only 22 studies that provided measures of interest (proportions or estimates of prevalence) and that adopted the Fried criteria to define frailty were included in the meta-analyses.

### Studies description

The studies sample sizes range from 62 to 6078 participants, with a mean age ranging from 52 to 88 years. Among 37 selected studies, 18 reported prevalence of frailty in clinical IHD patients with no comparison with persons without the disease (see Additional file 1: Appendix, Table 1c for description of selected studies) [21–38]. Most studies included community-dwelling participants, although all the studies that assessed frailty among populations of IHD patients included in-hospital participants.

Among the excluded studies, only two studies assessed frailty with the same scale (Canadian Study of Health and Aging Clinical Frailty Scale 7-items). Frailty prevalence in IHD patients ranged from 23.9 to 48.5%.

### IHD and frailty definition

Most of the studies (*n* = 24) defined frailty according to the Cardiovascular Health Study (CHS) criteria, proposed by Fried and colleagues [4]. An adaptation of the Fried criteria was used in one study. Other operational definitions for frailty were based on the use of the Edmonton Frail Scale, the Canadian Study of Health and Ageing’s clinical frailty scale, the SHARE-FI index, the Tilburg Frailty Indicator or by a composite frailty index [39–41]. One study assessed frailty adopting both Fried criteria and the Rockwood Frailty score and one study used both Fried and Green criteria [42, 43].

Detailed information regarding the operational definition and identification of IHD among studies participants is reported in Additional file 1:Table 1a, b and c in the Appendix. Among inpatient participants, the presence of IHD was ascertained quite rigorously as it was based on medical charts, physician’ judgment and clinical evaluation and following recognized international guidelines for IHD diagnosis. Among community dwelling participants, the identification of IHD was based on self-report in most cases.

### Association between frailty and IHD

**Fig. 2:**
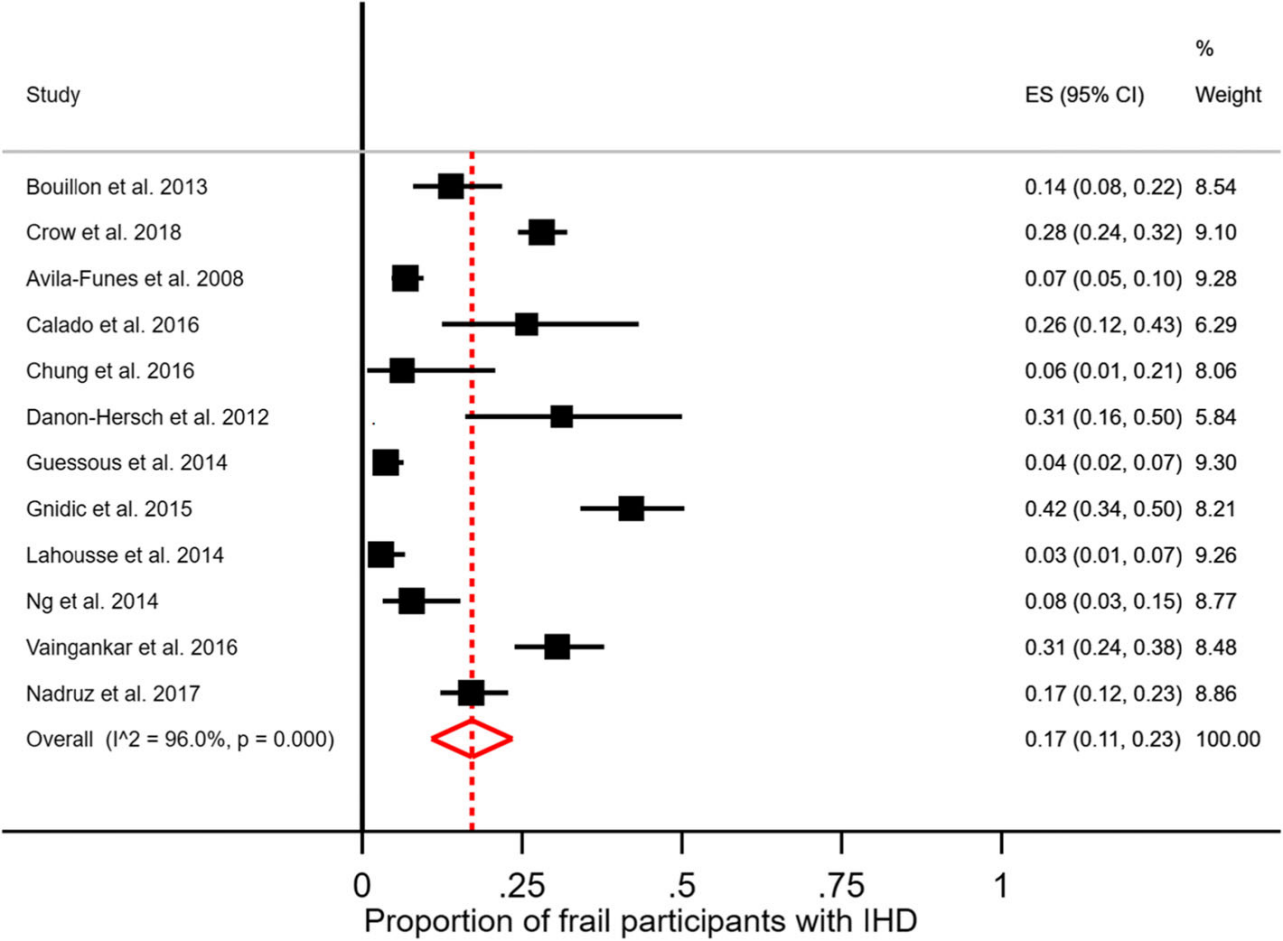
Proportion of frail participants with IHD

**Fig. 3:**
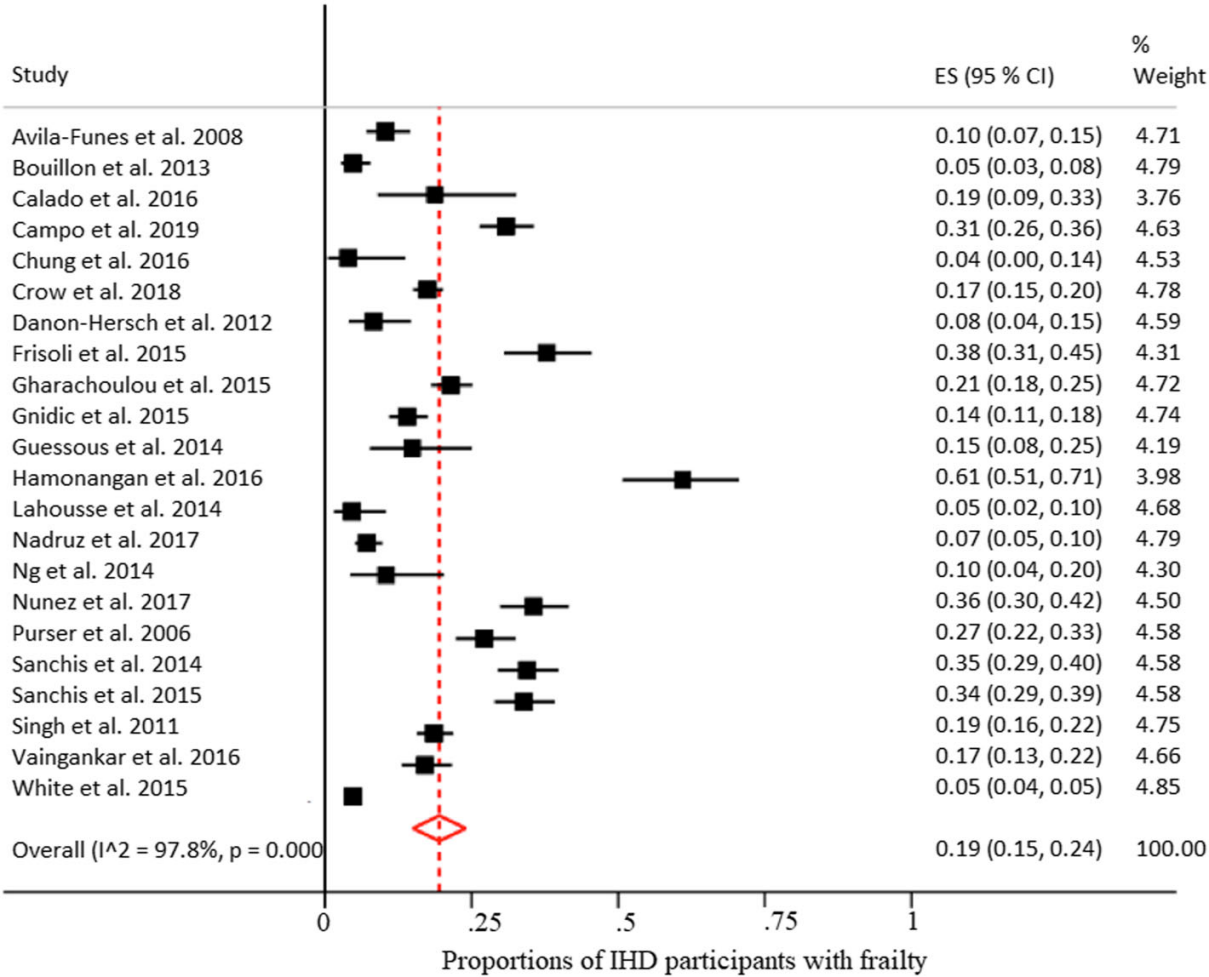
Proportion of IHD participants with frailty

The overall prevalence of IHD among frail participants was 17% (95% CI 11–23%) from the pooled analysis with estimates ranging from 3 to 42% (Fig. 2). The overall prevalence of frailty among participants with IHD was 19% (95%CI 15–24%) from the pooled analysis (Fig. 3) with estimates ranging from 4 to 61%. In studies including only participants with IHD, the prevalence of frailty ranged from 12 to 61%. After repeating the analyses excluding studies with < 500 participants we found comparable results, i.e., for the analysis reported in Fig. 2 a proportion of frail participants with IHD of 17% (95%CI 10–23); for the analysis reported in Fig. 3 a proportion of IHD participants with frailty of 11% (95%CI 8–15).

### Cross- sectional data

Twelve studies providing cross-sectional data of frailty and IHD were included in the systematic review (Additional file 1: Table 1a in the Appendix*)* [7, 21–41, 44]. Most of these studies defined frailty according to Fried criteria [4]. All these studies except one provided adequate estimates for the analyses of prevalence. Frailty resulted not associated with IHD in three studies but data from the reported multivariate models were not shown. Only two studies reported cross-sectional associations between frailty and IHD. Guessous and colleagues found that neither the presence of 1 frailty indicator nor the presence of 2 or more frailty indicators compared with the robust condition were associated with myocardial infarction (MI) (OR 0.96; 95%CI 0.54–1.71, and OR 0.78; 95% CI 0.37–1.64, respectively) [44]. Kleipool and colleagues reported an adjusted OR of MI for frailty 2.43; 95% CI 0.57–10.39 from the baseline data of their longitudinal study [21].

### Longitudinal data

Seven longitudinal studies of frailty and IHD were included in the systematic review (Additional file 1: Table 1b in the Appendix).(58-63) Of these, only two studies provided measures of the association between IHD and incident frailty defined according to Fried criteria. Bouillon and colleagues found that being at risk of IHD, defined according to the Framingham score for coronary artery disease (CHD), was associated with 38% increased risk of developing frailty in a sample of 3895 adult individuals free from cardiovascular disease at baseline and followed over 10 years [22]. In contrast, with such findings, Kleipool and colleagues found no association between IHD and frailty in a sample of 1432 older adults followed over 17 years,) (adj. HR of MI for frailty 0.58; 95% CI 0.14–2.32) [21]. Crow and colleagues analyzed data from the National Health and Nutrition Examination Survey (NHANES) and found a statistically significant increased risk of cardiovascular death associated with frailty (defined according to Fried criteria), after adjusting for socio-demographic variables and comorbidities (adj. HR 3.39; 95% CI 2.45–4.70) [21]. Similarly, another longitudinal study by Wallace and colleagues estimated a statistically significant increased risk of IHD events (HR 1.61; 95% CI 1.40–1.85) associated with frailty over 10 year follow-up. However, in this study a composite Frailty index was adopted to identify frailty [24]. The remaining three longitudinal studies adopted broad definitions of cardiovascular disease (CVD) that included IHD and several other heart conditions. Among these studies, Lee and colleagues found no significant association between heart disease and change in frailty status over 2 years, either in terms of worsening frailty or improvement [25]. In contrast with such results, Trevisan and colleagues found an almost twofold significant risk of progressing from prefrail to frail in persons with coronary disease over 4.4 years [26]. Pre-frailty (defined as the presence of 1 or 2 Fried criteria) was also found to be associated with an increased risk of developing CVD events (adj. HR 1.25; 95% CI 1.05–1.64, for people who met 1 Fried criterion; adj. HR 1.79; 95% CI 1.27–2.52, for people who met 2 Fried criteria) [27]. In this study, it was not possible to disentangle the specific effect of pre-frailty on IHD because a broad definition of CVD was adopted to identify the outcome.

### Assessment of risk of bias

The majority of the studies presented a moderate risk of bias according to the NOS. In most of the cases, the self-reported nature of information was responsible for low scores. Finally, a marginal evidence of publication bias was detected in our meta-analyses (Begg’s test *P* = 0.116 and Egger’s test *P* = 0.016).

## Discussion

This systematic review and meta-analysis shows that nearly one fifth of frail adults present with IHD and the same proportion of IHD adults present with frailty. Insufficient data were found to assess the longitudinal association between frailty and IHD.

As the population ages worldwide, an increasing number of older adults are expected to suffer from frailty and chronic cardiovascular disease [28, 29]. A link between cardiovascular risk and frailty has been frequently suggested. For example, a recent systematic review estimated that over 70% of frail adults present with hypertension and 14% of persons with hypertension also suffer from frailty [13]. Consistent with these data, the likelihood of the association between hypertension and frailty was 33% from the pooled analysis conducted in the same review, although such estimate was not statistically significant. Similarly, frailty is highly prevalent among adults with heart failure affecting up to half of such population [30]. The prevalence of frailty in patients with heart failure seems to increase with age reaching the estimate of 70% among persons over 80 years of age [32] Finally, there is some evidence that CVD risk may predict the risk of incident frailty, independently of the algorithm that is used to measure CVD risk score [22].

To date, evidence to systematically assess the association between frailty and IHD is lacking, and the available data are inconsistent. Also, the direction of the postulated association between these two conditions is unclear. Indeed, we found that very few longitudinal studies have assessed the impact of frailty on IHD and vice versa, and they have led to conflicting findings. It is likely that IHD may contribute to the development of frailty, as well as frailty may influence the course and prognosis of IHD in older adults. For example, older IHD patients may experience reduced tolerance to exercise because of chest pain or shortness of breath and this may in turn lead to physical inactivity, reduced muscle mass and strength (i.e. sarcopenia) and increased risk of frailty. On the other side, it is well known that frailty is associated with sarcopenia, cognitive and functional decline and multimorbidity [2]. Such conditions may actually determine low mobility and imbalance in body composition (overweight and obesity) thus negatively impacting on the CVD status and worsening the prognosis for IHD patients.

According to the current understanding of pathophysiology of IHD, atherosclerosis is considered a systemic inflammatory disease. In particular, a chronic increase of plasma levels of pro-inflammatory cytokines such as interleukins IL-6, IL-1β, IL-17, and TNF-α is known to characterize cardiovascular diseases including IHD. Such chronic inflammation represents a prolonged response to physical, psychological and environmental stressors and is believed to cause a progressive damage to cardiovascular tissues and to promote endothelial activation [32]. A low grade chronic inflammation and immune activation has been also demonstrated to play a central role in the pathophysiology of frailty [33, 34]. Advanced age, multiple chronic diseases, metabolic, psychosocial and environmental stressors contribute to chronic inflammation in frail individuals [4]. Inflammation produces detrimental effects on skeletal muscle promoting apoptosis and in turn leading to sarcopenia [35]. The neuro-endocrine system is also impaired by chronic inflammation and this, beyond boosting the risk of developing CVD, leads to several systemic effects such as loss of appetite, poor nutrition, weight loss and metabolic changes, ultimately resulting in reduced muscle mass and strength and increased body fat. Such changes may promote physical inactivity and contribute to osteoporosis and risk of falls. The above-mentioned changes may increase the vulnerability to stressors thus creating a vicious circle that sustains frailty and increases the risk of disability. These considerations suggest that frailty and IHD may share common pathophysiological pathways and Inflammation may represent the biological link between them.

There is growing awareness of frailty in cardiovascular medicine. According to international recommendations, frailty should be taken into account especially in the therapeutic decision making process [36, 37]. Frail patients are usually excluded from randomized clinical trials (RCT) populations and thus current recommendations for the treatment of IHD may not be applied to them [38]. Despite this lack of evidence, frailty is thought to influence therapeutic choices and outcomes. For example, it has been shown that frail older adults with acute coronary syndrome are less likely to receive intensive care compared with robust patients [39]. Also, frailty has been associated with prolonged hospital stay and recurrent hospitalizations for cardiovascular events in patients with acute MI.(43, 44) Negative clinical outcomes including death have been reported in frail older patients who were treated with percutaneous coronary intervention (PCI) [45, 46]. Regarding elderly with ACS, frailty was found to be associated to higher all-cause mortality in three recent metanalyses [16–18]. Anyway, the pooled analyses were conducted without stratification for the different definitions of frailty of the single studies and the estimation could be partly biased. Moreover, our study was not intended to be focused on ACS but our search strategy was able to find studies regarding elderly with frailty and ACS that were not included in those metanalyses.

With respect to pharmacological treatment, frailty may influence prescribing decisions especially in those patients who present with multiple chronic diseases and are at high risk of medications adverse reactions (ADRs) such as bleeding, hypotension, falls and acute renal failure [47]. Lower rates of angiotensin converting enzyme inhibitors (ACEi), angiotensin receptor blockers (ARB) and statins prescriptions following an acute coronary event have been reported in frail patients compared to robust individuals [41]. The lack of scientific evidence regarding frail patients with cardiovascular disease, the increased risk of adverse events and the limited life expectancy of these individuals, make extremely complex to assess the benefit/risk ratio associated with the available therapeutic options. Future cardiovascular RCTs should provide evidence for such high-risk population and measures of frailty should be used to stratify patients in these studies.

To our knowledge this is the first systematic review that specifically assessed the association between frailty and IHD. A comprehensive literature search and a careful selection and evaluation of studies have been performed to provide the best synthesis of the evidence in the field. However, some limitations of the present review need to be acknowledged. We found a significant heterogeneity among studies especially with respect of frailty and IHD operational definitions and characteristics of study populations. In particular, IHD was poorly defined in most studies including community-dwelling individuals, being based on patients’ self-report. Moreover, although some measures of association were available from the included studies and were reported in the present review, no study was found that specifically assessed the association between frailty and IHD. Despite our search strategy was meant to include as many IHD definitions as possible, some studies might have been excluded. The paucity of data retrieved, the heterogeneity of both frailty and IHD definitions and type of outcome evaluated in such studies (e.g., incident frailty, transition from pre-frail to frail status, incident CVD events, death) made it impossible to perform meta-analyses of the association between IHD and frailty.

## Conclusions

The present review and meta-analysis found that frailty is quite common in persons with IHD affecting nearly one fifth of them. The identification of frailty among patients with IHD should be considered relevant especially among older adults to identify appropriate outcomes of treatment and provide individualized strategies of cardiovascular prevention and care. Future studies should be designed to specifically investigate the association between frailty and IHD, both in chronic and in acute manifestations, and to explore the reciprocal influence of these two conditions on each other on both a biological and a clinical ground.

## Supplementary Information

**Additional file 1.**

## Data Availability

The datasets used and/or analysed during the current study are available from the corresponding author on reasonable request.

## Abbreviations

95% CI: 95% Confidence interval
ACEi: Angiotensin-converting enzyme inhibitors
ARB: Angiotensin receptor blockers
Adj: Adjusted
ADRs: Adverse drug reactions
CVD: Cardio-vascular diseases
HR: Hazard ratio
IHD: Ischemic heart disease
IL-1β: Interleukin 1 beta
IL-6: Interleukin 6
IL-17: Interleukin 17
MI: Myocardial infarction
NOS: Newcastle-Ottawa scale
OR: Odds ratio
PCI: Percutaneous coronary intervention
RCTs: Randomized controlled trials
TNF-α: Tumor necrosis factor alpha

## References

[1] Hoogendijk EO, Afilalo J, Ensrud KE, Kowal P, Onder G, Fried LP (2019). Frailty: implications for clinical practice and public health. Lancet.

[2] Clegg A, Young J, Iliffe S, Rikkert MO, Rockwood K (2013). Frailty in elderly people. Lancet.

[3] Dent E, Kowal P, Hoogendijk EO (2016). Frailty measurement in research and clinical practice: a review. Eur J Intern Med..

[4] Fried LP, Tangen CM, Walston J, Newman AB, Hirsch C, Gottdiener J (2001). Frailty in older adults: evidence for a phenotype. J Gerontol A Biol Sci Med Sci.

[5] Theou O, Cann L, Blodgett J, Wallace LM, Brothers TD, Rockwood K (2015). Modifications to the frailty phenotype criteria: systematic review of the current literature and investigation of 262 frailty phenotypes in the survey of health, ageing, and retirement in Europe. Ageing Res Rev.

[6] Collard RM, Boter H, Schoevers RA, Oude Voshaar RC (2012). Prevalence of frailty in community-dwelling older persons: a systematic review. J Am Geriatr Soc.

[7] Kojima G (2015). Prevalence of frailty in nursing homes: a systematic review and meta-analysis. J Am Med Dir Assoc.

[8] Lahousse L, Maes B, Ziere G, Loth DW, Verlinden VJA, Zillikens MC (2014). Adverse outcomes of frailty in the elderly: the Rotterdam study. Eur J Epidemiol.

[9] Veronese N, Cereda E, Stubbs B, Solmi M, Luchini C, Manzato E (2017). Risk of cardiovascular disease morbidity and mortality in frail and pre-frail older adults: results from a meta-analysis and exploratory meta-regression analysis. Ageing Res Rev.

[10] Stewart R (2019). Cardiovascular disease and frailty: what are the mechanistic links?. Clin Chem.

[11] Bellumkonda L, Tyrrell D, Hummel SL, Goldstein DR (2017). Pathophysiology of heart failure and frailty: a common inflammatory origin?. Aging Cell.

[12] Bouillon K, Kivimäki M, Hamer M, Shipley MJ, Akbaraly TN, Tabak A (2013). Diabetes risk factors, diabetes risk algorithms, and the prediction of future frailty: The whitehall II prospective cohort study. J Am Med Dir Assoc.

[13] Yang X, Lupón J, Vidán MT, Ferguson C, Gastelurrutia P, Newton PJ (2018). Impact of frailty on mortality and hospitalization in chronic heart failure: a systematic review and meta-analysis. J Am Heart Assoc.

[14] Vetrano DL, Palmer KM, Galluzzo L, Giampaoli S, Marengoni A, Bernabei R (2018). Hypertension and frailty: a systematic review and meta-analysis. BMJ Open.

[15] Villani ER, Tummolo AM, Palmer K, Gravina EM, Vetrano DL, Bernabei R (2018). Frailty and atrial fibrillation: a systematic review. Eur J Intern Med.

[16] Dou Q, Wang W, Wang H, Ma Y, Hai S, Lin X (2019). Prognostic value of frailty in elderly patients with acute coronary syndrome: a systematic review and meta-analysis. BMC Geriatr.

[17] Man C, Xiang S, Fan Y (2019). Frailty for predicting all-cause mortality in elderly acute coronary syndrome patients: a meta-analysis. Ageing Res Rev.

[18] Xu W, Cai Y, Liu H, Fan L, Wu C (2020). Frailty as a predictor of all-cause mortality and readmission in older patients with acute coronary syndrome : a systematic review and meta-analysis. Wien Klin Wochenschr.

[19] Liberati A, Altman DG, Tetzlaff J, Mulrow C, Gøtzsche PC, Ioannidis JPA (2009). The PRISMA statement for reporting systematic reviews and meta-analyses of studies that evaluate health care interventions: explanation and elaboration. J Clin Epidemiol.

[20] Stang A (2010). Critical evaluation of the Newcastle-Ottawa scale for the assessment of the quality of nonrandomized studies in meta-analyses. Eur J Epidemiol.

[21] Kleipool EEF, Hoogendijk EO, Trappenburg MC, Handoko ML, Huisman M, Peters MJL (2018). Frailty in older adults with cardiovascular disease: cause, effect or both?. Aging Dis.

[22] Bouillon K, Batty GD, Hamer M, Sabia S, Shipley MJ, Britton A (2013). Cardiovascular disease risk scores in identifying future frailty: the Whitehall II prospective cohort study. Heart..

[23] Crow RS, Lohman MC, Titus AJ, Bruce ML, Mackenzie TA, Bartels SJ (2018). Mortality risk along the frailty Spectrum: data from the National Health and nutrition examination survey 1999 to 2004. J Am Geriatr Soc.

[24] Wallace LMK, Theou O, Kirkland SA, Rockwood MRH, Davidson KW, Shimbo D (2014). Accumulation of non-traditional risk factors for coronary heart disease is associated with incident coronary heart disease hospitalization and death. PLoS One.

[25] Lee JSW, Auyeung TW, Leung J, Kwok T, Woo J (2014). Transitions in frailty states among community-living older adults and their associated factors. J Am Med Dir Assoc.

[26] Trevisan C, Veronese N, Maggi S, Baggio G, Toffanello ED, Zambon S (2017). Factors influencing transitions between frailty states in elderly adults: the Progetto Veneto Anziani longitudinal study. J Am Geriatr Soc.

[27] Sergi G, Veronese N, Fontana L, De Rui M, Bolzetta F, Zambon S (2015). Pre-frailty and risk of cardiovascular disease in elderly men and women: the pro.V.a. study. J Am Coll Cardiol.

[28] Afilalo J, Alexander KP, Mack MJ, Maurer MS, Green P, Allen LA (2014). Frailty assessment in the cardiovascular care of older adults. J Am Coll Cardiol.

[29] Benjamin EJ, Muntner P, Alonso A, Bittencourt MS, Callaway CW, Carson AP (2019). Heart disease and stroke Statistics-2019 update: a report from the American Heart Association. Circulation..

[30] Denfeld QE, Winters-Stone K, Mudd JO, Gelow JM, Kurdi S, Lee CS (2017). The prevalence of frailty in heart failure: a systematic review and meta-analysis. Int J Cardiol.

[31] Vidán AT, Sánchez E, Fernández-Avilés F, Serra-Rexach JA, Ortiz J, Bueno H (2014). FRAIL-HF, a study to evaluate the clinical complexity of heart failure in nondependent older patients: rationale, methods and baseline characteristics. Clin Cardiol.

[32] Fatkhullina AR, Peshkova IO, Koltsova EK (2016). The role of cytokines in the development of atherosclerosis. Biochemistry (Mosc).

[33] Walston J, McBurnie MA, Newman A, Tracy RP, Kop WJ, Hirsch CH (2002). Frailty and activation of the inflammation and coagulation systems with and without clinical comorbidities: results from the cardiovascular health study. Arch Intern Med.

[34] Phan HM, Alpert JS, Fain M (2008). Frailty, inflammation, and cardiovascular disease: evidence of a connection. Am J Geriatr Cardiol.

[35] Muscaritoli M, Anker SD, Argilés J, Aversa Z, Bauer JM, Biolo G (2010). Consensus definition of sarcopenia, cachexia and pre-cachexia: Joint document elaborated by Special Interest Groups (SIG) “ cachexia-anorexia in chronic wasting diseases” and “ nutrition in geriatrics.”. Clin Nutr.

[36] Ponikowski P, Voors AA, Anker SD, Bueno H, Cleland JGF, Coats AJS (2016). ESC guidelines for the diagnosis and treatment of acute and chronic heart failure: the task force for the diagnosis and treatment of acute and chronic heart failure of the European Society of Cardiology (ESC) developed with the special contribution of the heart failure association (HFA) of the ESC. Eur Heart J.

[37] Walker DM, Gale CP, Lip G, Martin-Sanchez FJ, McIntyre HF, Mueller C (2018). Editor’s choice - frailty and the management of patients with acute cardiovascular disease: a position paper from the acute cardiovascular care association. Eur Heart J Acute Cardiovasc Care.

[38] Rich MW, Chyun DA, Skolnick AH, Alexander KP, Forman DE, Kitzman DW (2016). Knowledge gaps in cardiovascular care of the older adult population. Circulation..

[39] Kang L, Zhang SY, Zhu WL, Pang HY, Zhang L, Zhu ML (2015). Is frailty associated with short-term outcomes for elderly patients with acute coronary syndrome?. J Geriatr Cardiol.

[40] Graham MM, Galbraith PD, O’Neill D, Rolfson DB, Dando C, Norris CM (2013). Frailty and outcome in elderly patients with acute coronary syndrome. Can J Cardiol.

[41] Patel A, Goodman SG, Yan AT, Alexander KP, Wong CL, Cheema AN (2018). Frailty and outcomes after myocardial infarction: insights from the CONCORDANCE registry. J Am Heart Assoc.

[42] Rockwood K, Song X, MacKnight C, Bergman H, Hogan DB, McDowell I (2005). A global clinical measure of fitness and frailty in elderly people. CMAJ..

[43] Green P, Woglom AE, Genereux P, Daneault B, Paradis JM, Schnell S (2012). The impact of frailty status on survival after transcatheter aortic valve replacement in older adults with severe aortic stenosis: a single-center experience. JACC Cardiovasc Interv.

[44] Guessous I, Luthi JC, Bowling CB, Theler JM, Paccaud F, Gaspoz JM (2014). Prevalence of frailty indicators and association with socioeconomic status in middle-aged and older adults in a swiss region with universal health insurance coverage: a population-based cross-sectional study. J Aging Res.

[45] Campo G, Maietti E, Tonet E, Biscaglia S, Ariza-Solè A, Pavasini R (2020). The assessment of scales of frailty and physical performance improves prediction of major adverse cardiac events in older adults with acute coronary syndrome. J Gerontol A Biol Sci Med Sci.

[46] Tse G, Gong M, Nunez J, Sanchis J, Li G, Ali-Hasan-Al-Saegh S (2017). Frailty and mortality outcomes after percutaneous coronary intervention: a systematic review and meta-analysis. J Am Med Dir Assoc.

[47] Palmer K, Villani ER, Vetrano DL, Cherubini A, Cruz-Jentoft AJ, Curtin D (2019). Association of polypharmacy and hyperpolypharmacy with frailty states: a systematic review and meta-analysis. Eur Geriatr Med.

