# Supplementary material for: Association between frailty and ischemic heart disease: a systematic review and meta-analysis
Source: BMC Geriatr. 2021 Jun 10;21:357. doi: 10.1186/s12877-021-02304-9 (PMC8193864; doi:10.1186/s12877-021-02304-9)
Supplement: Supplementary file 1 — Additional file 1. [file 12877_2021_2304_MOESM1_ESM.docx]

**APPENDIX 1**

**SEARCH STRATEGY**

**For pubmed**

(“coronary artery disease”[MeSH Terms] OR “coronary artery disease”[Title/Abstract] OR “ischemic heart disease”[Title/Abstract] OR “angina”[Title/Abstract] OR “coronary heart disease”[Title/Abstract] OR “myocardial infarction”[Title/Abstract] OR “coronaropathy”[Title/Abstract] OR “heart infarction”[Title/Abstract]) AND ("frail elderly"[MeSH Terms] OR "frail*"[Title/Abstract] OR "frailty"[Title/Abstract])

**For web of science and Embase**

(“coronary artery disease”  OR “ischemic heart disease”  OR “angina”  OR “coronary heart disease”  OR “myocardial infarction”  OR “coronaropathy”  OR “heart infarction” ) AND ("frail*"  OR "frailty")

**Table 1a. Characteristics of the studies included in the systematic review. Cross-sectional data**

| **First author**  **(year)** | **Study characteristics** | **n** | **IHD definition** | **Overall IHD incidence or prevalence (%)** | **Frailty definition** | **Overall Frailty incidence or prevalence (%)** | **IHD prevalence in frailty groups (%)** | **Estimates of association between IHD and Frailty** | **NOS** |
| --- | --- | --- | --- | --- | --- | --- | --- | --- | --- |
| Ávila-Funes et al., 2008 | Country: Switzerland  Name: Three-City Study  Setting: Community  Mean age ± sd: 74.1 ± 5.2 | 6,078 | Participants were asked whether they had a physician’s diagnosis of MI | 4.6% | Fried et al’s Criteria | Frailty=7%, Prefrailty=47.6% | Robust=3.9%  Prefrail=5%  Frail=6.9% | - | 6 |
| Calado et al.,  2016 | Country: Brazil  Name: the FIBRA study  Setting: Community, Independent living  Mean age ± sd: 73.9 ± 6.5 | 385 | Self-reported chronic diseases that had been recognized by a doctor during the past year as “heart disease”. | 12.5% | Fried Criteria | Rrailty=9.1%, Prefrailty=49.6% | Robust=9.4%  Prefrail=12.6%  Frail=25.7% | - | 6 |
| Chung et al.,  2016 | Country: Taiwan  Name: I-Lan Longitudinal Aging Study  Setting: Community  Mean age ± sd: 62.5 ± 8.6 | 962 | Coronary artery disease was identified by history taking. | 5.2% | Fried Criteria | - | Robust=4.9%  Prefrail=5.6%  Frail=6.3% | - | 7 |
| Danon-Hersch et al.,  2012 | Country: Switzerland  Name:Lausanne Cohort Lc65+,  Setting: Community  Age: older than 65 years of age | 1,283 | CHD was a self-reported medical diagnoses: “Has a doctor ever told you that you had CHD?” | 9.4% | Adjusted Fried criteria | Frailty=2.5%, Prefrailty=26.4% | Robust=7.9%  Prefrail=11.5%  Frail=31.3% | - | 7 |
| Espinoza et al., 2015 | Country: USA  Name: San Antonio Longitudinal Study of Aging (SALSA)  Setting: Community  Age range 65-80 years | 394 | Unspecified clinical measures were used to assess myocardial infarction | 2.5% | Fried criteria | Frailty=10.7% | - | It is reported in text that odds of frailty in MI were not significant in multiadjusted models (data not shown). | 5 |
| Guessous et al., 2014 | Country: Switzerland    Name: BusSante ́study,  Setting: community  Mean age: 60 years (SE 0.1) | 2,930 | Self-report: “Have you ever been told that you had myocardial infarction?” | - | Fried criteria | - | Robust=2.1%  Prefrail=2.9%  Frail=3.6% | Association between MI and 1 frailty indicator vs 0: adjusted. OR=0.96,95%CI 0.54-1.71. Association between MI and 2+ frailty indicators vs 0 adjusted OR=0.78, 95% CI 0.37-1.64. | 6 |
| Gnjidic et al.,  2015 | Country: Australia  Name: Concord Health and Ageing in Men Project  Setting: Community  Mean age ± sd: 77 ± 5.5 | 1,694 | IHD was considered present if a person reported having angina or a history of myocardial infarct/ACS/heart attack | 27% | Fried criteria. | Overall frailty 9% | - | - | 5 |
| Lahousse et al., 2014 | Country: The Netherlands  Name: Rotterdam study  Setting: Community  Median age 74 | 2,833 | MI was “clinically validated”. Coronary revascularization was defined as coronary artery bypass grafting and percutaneous coronary intervention. | - | Fried Criteria | Frailty=6%, Prefrailty=51% | Robust=2.4%  Prefrail=5.2%  Frail=3.1% | - | 7 |
| Nadruz et al., 2017 | Country: United States  Name: Atherosclerosis Risk in Communities (ARIC) Study  Mean age ± sd: 75.6±5.0 years | 3,991 | Coronary artery disease was defined as previous MI or coronary intervention. | 12.5% | Fried Criteria | Frailty =5.3% | Frail =17.1%  Robust= 12.3% | - | 7 |
| Ng et al.,  2014 | Country: Singapore  Name: SLAS – Singapore Longitudinal Ageing Studies I and II  Setting: Community  Mean age ± sd: 66.7 ± 7.8 | 1,685 | The self-report of a medical disorder diagnosed and treated by a physician(s) was recorded for 22 named diagnoses and other disorders. The presence of “coronary disease” supported by examination of medications used, physical examination or blood tests, electrocardiogram, fasting blood glucose, or history of coronary reperfusion procedures. | 3.9% | Fried Criteria | Frailty=5%, Prefrailty=42% | Robust=3.2  Prefrail=4.5  Frail=7.8 | It is reported in the text that the association between frailty and coronary disease was not significant (data not shown). | 6 |
| Vaingankar et al., 2016 | Country: Singapore  Name:Well-being of the Singapore Elderly study,  Setting: Community  Mean age 69 years | 2,102 | Field interviewers collected data on medical conditions. “Heart trouble or angina.” | 11.1% | Fried Criteria | Frailty=5.7%, Prefrailty=40.1% | Robust=9%  Prefrail=11.8%  Frail=11.8% | It is reported in the text that adjusted frailty and prefrailty ORs for heart trouble or angina were not significant (data not shown), | 6 |
| Watanabe et al., 2017 | Country: Japan  Name: Obu Study of Health Promotion for the Elderly (OSHPE),  Setting: Community  Mean age: 71 years | 4,720 | Self-reported “heart disease” on medical history questionnaire or face-to-face interview. | .11.3% | Limitations in 5 domains: mobility, strength, endurance, physical activity, and nutritional status 3-5 domains impaired= frail, 1-2=prefrail, 0=robust. | Frailty=11.3%, Prefrailty=57% | Robust=12.9  Prefrail=16.9  Frail=23.9 | - | 6 |

**Table 1b. Characteristics of the studies included in the systematic review. Longitudinal data.**

| **First author**  **(year)** | **Study characteristics** | **n** | **IHD definition** | **Overall IHD incidence or prevalence (%)** | **Frailty definition** | **Overall frailty incidence or prevalence (%)** | **IHD prevalence in frailty groups (%)** | **Estimates of association between IHD and Frailty** | **NOS** |
| --- | --- | --- | --- | --- | --- | --- | --- | --- | --- |
| Bouillon et al. 2013a | Country: UK  Name: Whitehall II study  Setting: Community  Mean age ± sd: 67.2 ± 6.0 | 3,895 | CVD risk factors, including HDL-Cholesterol, BP, antihypertensive medication, smoking, Diabetes, atrial fibrillation. Plus 4 CVD risk scores (Framingham, CHD, stroke prediction models and SCORE) | incident CVD at follow-up 8% | Fried criteria | Frailty=2.8% Prefrailty=37.1% | Robust=7.6  Prefrail=8.4  Frail=13.9 | OR per one sd increment in Framingham CHD risk score for future frailty = 1.38; 95% CI 1.20-1.59, during the 10 year follow-up. | 7 |
| Crow et al., 2018 | Country: United States  Name: National Health and Nutrition Examination Survey (NHANES) 1999–2004; National Death Index database  Setting: Community  Mean age ± sd: 71.1 ± 0.19 | 4,984 | Self-reported coronary artery disease, if participants answered the question “Has a doctor ever told you have coronary artery disaese?” | 18.3% | Fried criteria | Frailty=10.8%  Prefrailty=44.0% | Robust=14.2  Prefrail=20.1  Frail=30.9 | - | 6 |
| Kleipool et al., 2018 | Country: The Netherlands  Name: Longitudinal Aging Study Amsterdam (LASA)  Setting: community  Mean age ± sd: 78.3 ± 5.9 (subjects with CVD); 75.4 ± 6.6 (subjects without CVD ) | 1,432 | Diagnosis based on the presence of at least two of the the following three criteria: 1) self-reported symptoms of AP or MI; 2) use od disease specific medications during the past 2 weeks prior assessment; 3)medical records of general practitioner. | Overall prevalence of CVD : 20%  Prevalence of IHD among subjects with CVD: AP: 56%  MI: 11% | Fried Criteria | Overall prevalence of Frailty: 15%  Incidence of frailty: 23% (subjects with CVD); 25% (subjects with AP) 15% (subjects with MI) | - | Adjusted OR of MI for frailty 2.43; 95% CI 0.57-10.39 from the baseline data.  Adjusted HR of MI for frailty= 0.58; 95% CI 0.14-2.32. | 7 |
| Lee et al., 2014 | Country: China, Hong Kong,  Setting : Community  Age: 65 years of age or older | 3,018 | Participants were asked whether they had ever been told by a physician that they had heart disease (IHD, congestive heart failure or angina). Medical diagnoses were cross-checked in the computerized medical system database of the Hong Kong Hospital Authority. Diagnoses were counted as present if reported by the participant or recorded in the medical database. | 19.0% in men; 16.7% in women | Fried criteria | Frailty  in men=6% in women=9.8%, Prefrailty  in men=48.7%  in women=52.2% | - | - | 7 |
| Sergi et al., 2015 | Country: Italy  Name: Progetto Veneto Anziani (Pro.V.A.)  Setting: Community  Mean age ± sd: 73.6 ± 6.7 | 1,567 | Coronary heart disease was defined as a history of revascularization, hospitalization for MI, electrocardiographic evidence of MI, or self-reported history of MI or angina, accompanied by the use of antianginal medication. CVD included CHD, heart failure, stoke, and peripheral artery disease. | - | Fried Criteria | - | - | - | 7 |
| Trevisan et al., 2017 | Country: Italy  Name: Progetto Veneto Anziani (Pro.V.A.)  Setting: Community  Mean age ± sd: 74.4 ± 7.3 | 2,925 | Based on personal interview, medical interview and clinical examination including blood tests. CVD was defined as atrial fibrillation; congestive heart failure; angina pectoris requiring a stent, angioplasty, or hospitalization; myocardial infarction; or stroke. |  | Fried Criteria | Frailty=6.6%, Prefrailty=49.3% |  | Significant risk of progressing from prefrailty to frailty associated with coronary disease (adjusted OR=1.96; 95% CI 1.7–2.2) | 7 |
| Wallace et al., 2014 | Country: Canada  Name: 1995 Nova Scotia Health Survey  Setting: Community  Mean age ± sd: 46.9 ± 18.7 | 2,195 | Based on ICD-9 diagnostic codes at hospital discharge. To ensure that CHD events were incident and not pre-existing, survey participants were asked about previous CHD events, and discharge diagnoses for each participant for 4 years prior to the baseline survey were reviewed. | 0% baseline. 8% incident CHD. | Frailty index including 17-self-reported variables unrelated to CHD (CHD-associated co-morbidities, ADL, health conditions such as glaucoma, arthritis, sinusitis, incontinence, and dependence for personal care or affairs), and 9 traditional risk factors for CHD (e.g. diabetes, hypertension). | Frailty index mean score 0.18 (corresponding to 4.5 of possible 25 deficits) |  | Frailty was associated with incident CHD events (adjusted HR=1.6; 95%CI 1.4–1.9). | 7 |

**Table 1c. Characteristics of the studies included in the systematic review. Studies including only patients with IHD.**

| **First author**  **(year)** | **Study characteristics** | **N** | **IHD definition** | **Overall IHD incidence or prevalence (%)** | **Frailty definition** | **Overall Frailty incidence or prevalence (%)** | **IHD prevalence in frailty groups (%)** | **NOS** |
| --- | --- | --- | --- | --- | --- | --- | --- | --- |
| Campo et al., 2019 | Country: Italy  Name: FRASER (Frailty in elderly patients receiving cardiac interventional procedures)  Setting: inpatients, hospitals  Mean age ± sd:  78.0 ± 6.0 | 402 | Hospital admission for ACS receiving coronary artery angiography ± PCI | 100 | Fried Criteria | Frailty= 31%  Prefrailty= 40%, | - | **8** |
| Ekerstad et al., 2011 | Country: Sweden  Setting: Inpatients, hospitals  Age: 75 years or olders | 307 | Patients with diagnosed NSTEMI according to their attending physicians. International Classification of Diseases, Ninth Revision (ICD-9). | 100 | Canadian Study of Health and Aging Clinical Frailty Scale (7 item). | Prefrailty (5 items)= 48.5%, Moderately or severely frail (6/7 items)= 24.1% | - | 5 |
| Frisoli et al.,  2015 | Country: Brazil  Name: Fragicor (FRAgilidade em idosos com doenças CardiOvasculaRes/Frailty in an older population with CVD),  Setting: Community  Mean age ± sd:  77.1 ± 5.9 | 172 | Diagnosis of Ischemic heart disease was made according to the American Heart Association (AHA) Guidelines. | 100 | Fried Criteria | Frailty= 39.8%,  Prefrailty= 51.5% | - | 5 |
| Gharacholou et al., 2012 | Country: United States  Setting: Inpatients, hospitals  Mean age ± sd:  74.3 ± 6.4 | 629 | Patients over 65 years of age undergoing percutaneous coronary intervention | 100 | Fried Criteria | Frailty=19%  Prefrailty=47% | - | 6 |
| Graham et al., 2013 | Country: Canada  Setting: Inpatients, hospitals  Age: 65 years or older | 183 | Patients aged 65 years or older with a diagnosis of ACS who were admitted over a period of 6 months to the cardiology inpatient unit at the University of Alberta Hospital | 100 | Edmonton Frail Scale (EFS): Scores range from 0 (not frail) to a maximum of 17 (very frail). | FS 0-3= 34.4%  EFS 4-6= 35.5%  EFS > 7= 30.1% | - | 7 |
| Hamonangan et al., 2016 | Country: Indonesia  Setting: Inpatients, hospitals  Mean age ± sd:  66.9 ± 4.9 | 100 | Patients who had been diagnosed with coronary heart disease who had undergone elective percutaneous coronary intervention. | 100 | Fried criteria | Frailty=61% | - | 7 |
| Kang et al.,  2015 | Country: China  Setting: hospital  Age: 65 years or older | 352 | Patients with diagnosed ACS according to their attending physicians, defined as a spectrum of conditions with non-ST elevation acute coronary syndromes (NSTE-ACS) and ST-elevation myocardial infarction (STEMI) according to the American College of Cardiology Foundation and American Heart Association. | 100 | Clinical Frailty Scale | Frailty=26.4%.  Prefrailty=43.2% |  | 7 |
| Lisiak et al.,  2016 | Country: Poland  Setting: Inpatients, hospitals  Mean age ± sd:  76.7 ± 7.8 | 91 | ACS diagnosed according to the European Society of Cardiology guidelines. | 100 | Tilburg frailty indicator | Global Frailty score mean: 7.4±2.6 | - | 6 |
| Lurie et al.,  2015 | Country: Israel ,  Name: Israel Study of First Acute Myocardial Infarction patients  Setting: inpatients at baseline; community follow-up  Mean age ± sd:  52.0± 8.6 | 558 | Patients admitted to hospital with incident MI. ICD9 for acute MI, including infarct type (Q-wave or non-Q-wave) and Killip class. | 100 | Adapted Mitnitski and Rockwood index of accumulation of deficits, a 40-item frailty index including: Perceived health , Comorbid conditions, Functional limitations, BMI, Physical Activity Scale for the Elderly, Mental health inventory. Scores were summed and divided by the total number of deficits to give a frailty index between 0 and 1, with 1 representing the greatest frailty. A threshold of 0.25 was used to define frailty. | 28% patients became frail at follow-up (10-13 years after the index MI) | - | 8 |
| Myers et al.,  2014 | Country: Israel ,  Name: Israel Study of First Acute Myocardial Infarction patients  Setting: inpatients at baseline; community follow-up  Mean age ± sd:  52.0± 8.6 | 1,151 | Patients admitted to hospital with incident MI. ICD9 for acute myocardial infarction, including infarct type (Q-wave or non-Q-wave) and Killip class. | 100 | Adapted Mitnitski and Rockwood index of accumulation of deficits, a 40-item frailty index including: Perceived health , Comorbid conditions, Functional limitations, BMI, Physical Activity Scale for the Elderly, Mental health inventory. Scores were summed and divided by the total number of deficits to give a frailty index between 0 and 1, with 1 representing the greatest frailty. A threshold of 0.25 was used to define frailty. | 34.7% became frail at follow-up (10-13 years after the index MI) | - | 7 |
| Núñez et al.,  2017 | Country: Spain  Setting: inpatients at baseline; community follow-up  Mean age ± sd:  78 ± 7 | 270 | Patients hospitalized with high-risk non-ST segment acute coronary syndromes | 100 | Fried Criteria | Frailty=35.6% | - | 7 |
| Purser et al.,  2006 | Country: United States  Setting: Inpatients, hospitals  Mean age ± sd:  77 ± 5 | 309 | Inpatients who underwent cardiac catheterization during their episode of care. Including two or more coronary vessels 75% or more stenosed, 50% or greater left main coronary artery occlusion, and prior (CABG) with one or more occluded grafts. | 100 | Phenotype Composite A: Fried Criteria (>3); Phenotype Composite B: Rockwood Frailty score (>1). | Frailty according to Fried criteria =27%  Frailty according to Rockwood criteria=63% | - | 6 |
| Salinas et al., 2016 | Country: Spain  Setting : inpatients, hospitals  Mean age ± sd:  Frail patients 83.8±5.7  Non frail patients 81.6±4.1 | 202 | Type 1 MI according to the American College of Cardiology/American Heart Association/European Society of Cardiology/World Heart Federation universal definition. | 100 | SHARE-FI index | Frailty=37.9%, Prefrailty=28.4% | - | 6 |
| Sanchis et al.,2014 | Country: Spain  Setting : inpatients, hospitals  Mean age ± sd:  77 ± 7 | 342 | The diagnosis of ACS was established by the chief concern of acute chest pain and any of the following criteria: (1) ST-segment elevation in the initial electrocardiogram suggestive of acute myocardial infarction, prompting reperfusion therapy, and confirmed with troponin elevation in the subsequent blood tests; (2) troponin elevation in the absence of ST-segment elevation, leading to the diagnosis of non–ST-segment elevation acute myocardial infarction; and (3) normal troponin with any of the following criteria of acute ischemia leading to unstable angina diagnosis: (a) ST-segment depression (N0.5 mm) or T-wave inversion (N2 mm) in the initial or subsequent electrocardiograms, (b) positive noninvasive stress test result (either exercise test or cardiac magnetic resonance with dypiridamole), and (c) significant stenosis in the coronary angiogram. | 100 | Frailty: Fried score ≥3; Green score ≥5. | Frailty Fried score frail=34%,  Frailty Green score frail=48% | - | 6 |
| Sanchis et al., 2015 | Country: Spain  Setting : inpatients, hospitals  Mean age ± sd:  Frail 81±7 Non frail 77±7 | 342 | Patients presented at the hospital with the chief complaint of acute chest pain leading to the diagnosis of ACS, including both ST-segment elevation and non-ST-segment elevation. Troponin elevation was observed in 92% of th patients (Elecsys hs-cTnT assay, Roche Diagnostics, Basel, Switzerland; myocardial infarction diagnosis cutoff14 ng/mL). In the remaining patients with normal troponin, the diagnosis of unstable angina was established by a positive noninvasive stress test (either exercise test or cardiac magnetic resonance with dipyridamole) or by the evidence of significant stenosis in the coronary angiogram. | 100 | Fried criteria | Frailty=33.9% |  | 6 |
| Singh et al.,  2011 | Country: United States  Setting: Inpatients, hospitals  Mean age ± sd:  74.3 ± 6.4 | 629 | Patients aged ≥ 65 undergoing percutaneous coronary intervention. | 100 | Fried criteria | Frailty=11.8%,  Prefrailty=47.4% | - | 8 |
| Sujino et al.,  2015 | Country: Japan  Setting: Inpatients, hospitals  Mean age ± sd:  88.1 ± 2.5 | 62 | STEMI was defined as: (1)clinical evidence of ischemia, (2) echocardiogram showing new ST elevation at the J-point in two contiguous leads, with a cut-off point of >0.2 mV in men and >0.15 mV in women in leads V2–V3 or >0.1 mV in the other leads, (3) at least one high myocardial biomarker level, defined as a serum troponin I or creatine kinase level above the 99th percentile of the normal reference population during the first 24 h after admission. | 100 | Canadian Study of Health and Aging Clinical Frailty Scale (CSHA-CFS). | Frailty = 35.5% | - | 5 |
| White et al.,  2016 | 52 countries  Name: TaRgeted platelet Inhibition to cLarify the Optimal strateGy to medicallY manage Acute Coronary Syndromes (TRILOGY ACS)  Setting:  community  Age: 65 years or older | 4,996 | High-risk patients with unstable angina who were managed medically and who were taking part in the TRILOGY ACS. | 100 | Fried Crtieria | Frailty=4.7%  Prefrailty=23% | - | 8 |

Abbreviations use in tables 1a, 1b, 1c:

ACS=acute coronary syndrome; CABG=coronary artery bypass graft ; CHD=coronary heart disease; CVD= cardiovascular disease; EFS= Edmonton Frail Scale; HR=hazard ratio; ICD-9=International Classifications of Diseases, 9^th^ Edition; IHD=ischemic heart disease; sd=standard deviation; MI=myocardial infarctio=Non-ST-elevation myocardial infarction; OR=odds ratio; PCI=percutaneous coronary intervention; SCORE=systematic coronary risk evaluation; SE= standard error; SES=socio-economic status; SHARE-FI index= Survey of Health, Ageing and Retirement in Europe Frailty Instrument; STEMI=ST-elevation myocardial infarction.

All results have been rounded to 1 decimal point
